# Molecular Mechanisms of the Diabetogenic Effects of Arsenic: Inhibition of Insulin Signaling by Arsenite and Methylarsonous Acid

**DOI:** 10.1289/ehp.9867

**Published:** 2007-01-29

**Authors:** David S. Paul, Anne W. Harmon, Vicenta Devesa, David J. Thomas, Miroslav Stýblo

**Affiliations:** 1 Department of Nutrition and; 2 Center for Environmental Medicine, Asthma, and Lung Biology, The University of North Carolina at Chapel Hill, Chapel Hill, North Carolina, USA; 3 Experimental Toxicology Division, National Health and Environmental Effects Research Laboratory, Office of Research and Development, U.S. Environmental Protection Agency, Research Triangle Park, North Carolina, USA

**Keywords:** arsenic, diabetes, glucose uptake, PDK-1, PKB/Akt

## Abstract

**Background:**

Increased prevalences of diabetes mellitus have been reported among individuals chronically exposed to inorganic arsenic (iAs). However, the mechanisms underlying the diabetogenic effects of iAs have not been characterized. We have previously shown that trivalent metabolites of iAs, arsenite (iAs^III^) and methylarsonous acid (MAs^III^) inhibit insulin-stimulated glucose uptake (ISGU) in 3T3-L1 adipocytes by suppressing the insulin-dependent phosphorylation of protein kinase B (PKB/Akt).

**Objectives:**

Our goal was to identify the molecular mechanisms responsible for the suppression of PKB/Akt phosphorylation by iAs^III^ and MAs^III^.

**Methods:**

The effects of iAs^III^ and MAs^III^ on components of the insulin-activated signal transduction pathway that regulate PKB/Akt phosphorylation were examined in 3T3-L1 adipocytes.

**Results:**

Subtoxic concentrations of iAs^III^ or MAs^III^ had little or no effect on the activity of phosphatidylinositol 3-kinase (PI-3K), which synthesizes phosphatidylinositol-3,4,5-triphosphate (PIP_3_), or on phosphorylation of PTEN (phosphatase and tensin homolog deleted on chromosome ten), a PIP_3_ phosphatase. Neither iAs^III^ nor MAs^III^ interfered with the phosphorylation of 3-phosphoinositide-dependent kinase-1 (PDK-1) located downstream from PI-3K. However, PDK-1 activity was inhibited by both iAs^III^ and MAs^III^. Consistent with these findings, PDK-1-catalyzed phosphorylation of PKB/Akt(Thr308) and PKB/Akt activity were suppressed in exposed cells. In addition, PKB/Akt(Ser473) phosphorylation, which is catalyzed by a putative PDK-2, was also suppressed. Notably, expression of constitutively active PKB/Akt restored the normal ISGU pattern in adipocytes treated with either iAs^III^ or MAs^III^.

**Conclusions:**

These results suggest that inhibition of the PDK-1/PKB/Akt-mediated transduction step is the key mechanism for the inhibition of ISGU in adipocytes exposed to iAs^III^ or MAs^III^, and possibly for impaired glucose tolerance associated with human exposures to iAs.

Arsenic (As) is a naturally occurring toxic metalloid and a potent human carcinogen [International Agency for Research on Cancer (IARC) 1987]. The cancer-promoting effects of environmental exposures to inorganic arsenic (iAs) have been examined by epidemiologic studies and in laboratory experiments. Much less attention has been paid to the adverse effects of iAs that do not involve malignancies. Epidemiologic evidence suggests that type 2 (noninsulin dependent) diabetes mellitus may be one of the most common noncancerous diseases associated with chronic exposures to iAs. Increased prevalences of type 2 diabetes or symptoms consistent with this disease have been associated with the consumption of drinking water containing high levels of iAs (Chen et al. 1995; Lai et al. 1994; Rahman et al. 1998, 1999; Tseng et al. 2000, 2002; Wang et al. 2003) or with chronic exposures to iAs in occupational settings (Jensen and Hansen 1998; Rahman and Axelson 1995; Rahman et al. 1996). Although not all epidemiologic studies support the association between iAs exposure and diabetes (Navas-Acien et al. 2006), the existing evidence provides sufficient basis for investigation of the diabetogenic effects of iAs.

Type 2 diabetes is characterized by disruptions in whole-body glucose homeostasis due to insulin resistance and impaired glucose utilization by peripheral tissues, including skeletal muscle and adipose tissue. The insulin-dependent activation of glucose uptake in these tissues is one of the key mechanisms that regulates glucose homeostasis. The insulin-activated signal transduction mechanism that stimulates glucose uptake by adipocytes has been extensively studied. It includes the autophosphorylation of the β-subunit of the insulin receptor (IRβ) upon binding of insulin to the α-subunit of the receptor (IRα), the subsequent tyrosine phosphorylation of insulin receptor substrate 1 or 2 (IRS-1 or -2), and the binding of a phosphorylated IRS (p-IRS) to the regulatory (p85) subunit of the class IA phosphatidylinositol 3-kinase (PI-3K) that leads to the activation of its catalytic (p110) subunit. The activated PI-3K catalyzes the phosphorylation of phosphatidylinositol-4,5-bisphosphate (PIP_2_) at the plasma membrane to phosphatidylinositol-3,4,5-triphosphate (PIP_3_) (Farese 2001; Ruderman et al. 1990; White and Kahan 1994). PIP_3_ facilitates 3-phosphoinositide-dependent kinase-1/2 (PDK-1/2) dependent phosphorylation/activation of protein kinase B (PKB/Akt) and two atypical enzymes of the protein kinase C (PKC) family, PKCλ and ζ (Chou et al. 1998; Le Good et al. 1998; Standaert et al. 1997). The phosphorylation of PKB/Akt results in the translocation of intracellular vesicles containing glucose transporter-4 (GLUT4) from the perinuclear region to the plasma membrane and in the stimulation of glucose uptake (Kohn et al. 1996a; Tanti et al. 1997). In addition to phosphorylated PKB/Akt (p-PKB/Akt), phosphorylated PKCλ (p-PKCλ), and PKCζ (p-PKCζ) are thought to participate in the stimulation of GLUT4 translocation in response to insulin signaling (Elmendorf and Pessin 1999; Ruderman et al. 1990). The mechanism by which p-PKB/Akt, p-PKCλ and ζ induce the translocation and fusion of GLUT4-containing vesicles with the plasma membrane, as well as the degree to which each of these kinases participates in this event, are unclear. Recent studies have indicated that the PI-3K-dependent rearrangement of actin filaments (Patel et al. 2003) and activation of the microtubule-associated motor protein kinesin (Imamura et al. 2003) contribute to the translocation of GLUT4 to the plasma membrane. The disruption of cytoskeletal components may represent a potential mechanism by which As exposure inhibits insulin-stimulated glucose uptake (ISGU). Notably, As has been shown to bind to actin and tubulin in human lymphoblastoid cells (Menzel et al. 1999) and to inhibit the cytoskeletal protein synthesis in Swiss 3T3 mouse cells (Li and Chou 1992).

The mechanisms by which exposure to iAs may induce impaired glucose tolerance have not been systematically studied. Data on the effects of As on glucose homeostasis have been generated almost exclusively in studies that examined the metabolism of nutrients under severe stress induced by chemical or physical stimuli. Results of *in vitro* studies have consistently shown significant increases in basal (insulin-independent) glucose uptake by various types of cells or dissected tissues exposed to cytotoxic concentrations of a trivalent iAs, arsenite (iAs^III^), or an aromatic derivative of As^III^, phenylarsine oxide (PAO) (Bazuine et al. 2003, 2004; Brazy et al. 1980; McDowell et al. 1997; Pasternak et al. 1991; Short 1965; Sviderskaya et al. 1996; Widnell et al. 1990). Consistent with these findings, some *in vivo* studies have reported moderate or severe hypoglycemia in animals chronically exposed to toxic, often lethal, concentrations of iAs^III^ or arsenate (iAs^V^), in drinking water (Hughes and Thompson 1996; Pal and Chatterjee 2004a, 2004b, 2005). Only limited information is available on the effects of arsenicals on glucose metabolism at low concentrations that are compatible with environmental or occupational exposures. Micromolar concentrations of PAO have been shown to inhibit basal or ISGU by cultured cells (Liebl et al. 1992, 1995) and by intact skeletal muscle (Henriksen and Holloszy 1990; Sowell et al. 1988). PAO did not interfere with the insulin-dependent phosphorylation of IRβ and did not interact directly with glucose transporters (Frost and Lane 1985; Frost et al. 1987). The effects of physiologically relevant arsenicals on insulin-stimulated glucose metabolism have only recently been examined in this laboratory (Walton et al. 2004). We have shown that iAs^III^ and the products of iAs methylation in humans, methylarsonous acid (MAs^III^), and dimethylarsinous acid (DMAs^III^) (Thomas et al. 2001), inhibit ISGU by 3T3-L1 adipocytes at concentrations that do not affect cell viability. Exposures to these arsenicals did not prevent IRβ and IRS phosphorylation or formation of the PI-3K–p-IRS complex. However, both iAs^III^ and MAs^III^ inhibited the insulin-dependent phosphorylation of PKB/Akt that mediates ISGU in adipocytes. In contrast, DMAs^III^ did not inhibit PKB/Akt phosphorylation, suggesting that this metabolite of iAs inhibits ISGU by a PKB/Akt-independent mechanism.

In the present study we examined the molecular mechanisms of ISGU inhibition by iAs^III^ and MAs^III^, focusing mainly on the components of the insulin-activated signal transduction pathway that regulate PKB/Akt phosphorylation in adipocytes. Results of this work show that iAs^III^ and MAs^III^ inhibit PDK-1 activity, thus suppressing PDK-1–catalyzed phosphorylation of PKB/Akt and p-PKB/Akt–mediated translocation of GLUT4 transporters to the plasma membrane. Notably, MAs^III^ was an order of magnitude more potent than iAs^III^ as an inhibitor of the PDK-1/PKB/Akt signal transduction step and of glucose uptake in insulin-stimulated adipocytes. Thus, the formation of MAs^III^ in the methylation pathway for iAs may play a critical role in determining the extent of the diabetogenic effects associated with chronic exposures to iAs.

## Materials and Methods

### Cell culture and treatment

We obtained 3T3-L1 preadipocytes from Y. Patel (University of North Carolina, Greensboro, North Carolina). Myr-PKB/Akt*–*3T3-L1 preadipocytes expressing constitutively active PKB/Akt lacking the pleckstrin homology (PH) domain were provided by S. Summers (University of Colorado at Boulder, Boulder, Colorado). Addition of the *src* myristoylation sequence promotes constitutive membrane association and activation of PKB/Akt (Kohn et al. 1996a, 1996b). A2myr-PKB/Akt–3T3-L1 adipocytes, which express PKB/Akt containing a nonfunctional *src* myristoylation domain, and 3T3-L1 adipocytes containing the empty expression vector were also provided by S. Summers. All cell lines were cultured in Dulbecco’s modified Eagle medium (Gibco, Grand Island, NY) with high glucose, 10% fetal bovine serum (HyClone, Logan, UT), penicillin, and streptomycin (Sigma Chemical Co., St. Louis, MO). Cells were cultured at 37°C in a humidified incubator in a 90% air and 10% CO_2_ atmosphere. To induce differentiation, postconfluent cells were treated with a mixture of 0.5 mM 3-isobutyl-1-methylxanthine, 1 μM dexamethasone, and 1 μg/mL insulin (all from Sigma Chemical Co.) for 48 hr and cultured in insulin-containing medium for an additional 48 hr (Paul et al. 2003). All experiments were performed between days 9 and 12 postinduction, when more than 90% of cells were fully differentiated. Differentiated adipocytes were treated with iAs^III^ (sodium salt, Sigma Chemical Co.) or methylarsine oxide (provided by W. Cullen, University of British Columbia, Vancouver, Canada). Identity and purity of methylarsine oxide was confirmed by ^1^H-NMR and mass spectrometry. In aqueous solutions, methylarsine oxide is hydrolyzed to form MAs^III^ (Petrick et al. 2001). Fresh stock solutions of iAs^III^ and MAs^III^ in sterile phosphate-buffered saline (PBS) were prepared before each experiment to minimize the oxidation of iAs^III^ to iAs^V^ or MAs^III^ to methylarsonic acid (MAs^V^). Adipocytes were incubated with arsenicals or vehicle in a cell culture incubator for 4 hr.

### Glucose uptake assay

The glucose uptake assay followed the previously described procedures (Paul et al. 2003). Briefly, adipocytes were serum starved in the presence or absence of arsenicals for 4 hr, washed with Krebs-Ringer phosphate (KRP) buffer, and treated with 1 μM insulin at 37°C for 10 min. Insulin-activated cells were incubated for 10 min with 200 μM 2-[1-^14^C]-deoxy-d-glucose (0.1 μCi/well) (NEN Life Science Products, Inc., Boston, MA). To measure basal (insulin-independent) glucose uptake, we incubated cells with radiolabeled glucose without pretreatment with insulin. After the incubation, cells were washed twice with PBS (0°C), and lysed in a solution of 0.5 N NaOH and 10% SDS. Radioactivity in cell lysates was measured, using a Wallac 1409 liquid scintillation counter (Wallac, Turku, Finland).

### Evaluation of cytotoxic and apoptotic effects of arsenicals

To determine cell viability, we used the MTT assay, which measures the conversion of 3-[4,5-dimethylthiazol-2-yl]-2,5-diphenyltetrazolium bromide (MTT) to purple formazan by mitochondrial dehydrogenases of viable cells (Carmichael et al. 1987), as previously described (Walton et al. 2004). Caspase-3 activity was examined in an assay mixture containing cell lysate and aminomethylcoumarin (AMC)-derived substrate, Z-DEVD-AMC (Molecular Probes, Carlsbad, CA). Cleavage of Z-DEVD-AMC by caspase-3 yields a blue-fluorescent product (excitation/emission wavelength = 342/441 nm) that was quantified by an HTS 7000 Bio Assay Reader (Perkin-Elmer, Norwalk, CT). We examined DNA fragmentation in adipocytes using TUNEL (terminal deoxynucleotidyltransferase-mediated nick end labeling). For this assay, adipocytes were cultured on glass coverslips coated with poly-l-lysine (Sigma Chemical Co.) and treated with arsenicals. Cells were then fixed in 4% buffered-paraformaldehyde and permeabilized in a solution of 0.1% Triton X-100 and 0.1% sodium citrate (Sigma Chemical Co.). DNA strand breaks were enzymatically labeled on 3′-OH termini with fluorescein-linked nucleotides, using the *In Situ* Cell Death Detection Kit (Roche Applied Science, Indianapolis, IN). Nuclei of both normal and apoptotic cells were stained with 100 nM 4′,6-diamidino-2-phenylindole dihydrochloride (DAPI) (Sigma Chemical Co.). Labeled cells were visualized using a Nikon Microphot FXA fluorescent microscope (Nikon, Tokyo, Japan).

### Immunofluorescent analysis of GLUT4

Adipocyte cultures on glass cover slips were treated with arsenicals, activated with insulin, and incubated with d-glucose (Sigma Chemical Co.). After fixation with 4% buffered-paraformaldehyde, adipocytes were rinsed with ice-cold PBS and incubated with poly-l-lysine (0.5 mg/mL) for 1 min. Cells were then treated with a hypotonic buffer [10 mM HEPES (pH 7.5), 2 mM MgCl_2_, 23 mM KCl, 1 mM EDTA] and pulse sonicated for 5 sec in a sonication buffer [30 mM HEPES (pH 7.5), 6 mM MgCl_2_, 70 mM KCl, 3 mM EGTA, 1 mM dithiothreitol, 0.1 mM phenylmethylsulfonyl fluoride (PMSF)], using a Fisher Model 100 Sonic Dismembrator equipped with a 12.7 × 1.3 × 0.3-cm probe (Fisher Scientific, Hampton, NH). Plasma membrane sheets attached to the coverslip were washed twice with the sonication buffer, incubated with an anti-GLUT4 antibody (Santa Cruz Biotech, Santa Cruz, CA) and labeled with Alexa-fluor 594 (Molecular Probes). Fluorescent images were captured, using a Zeiss LSM 110 fluorescent microscope (Zeiss, Jena, Germany).

### Speciation analysis of As

We analyzed arsenicals in cell cultures exposed to iAs^III^ or MAs^III^ using hydride generation atomic absorption spectrometry (HG-AAS) as previously described (Devesa et al. 2004). Cells and culture medium were analyzed separately for each treatment. Arsines were generated at pH 1, cold-trapped, separated by their boiling points, and analyzed, using a Perkin-Elmer model 5100 PC atomic absorption spectrometer (Perkin-Elmer). Under these conditions, arsines were generated from both As^III^ and As^V^ species. Thus, total iAs (iAs = iAs^III^ + iAs^V^), total methylarsenic (MAs = MAs^III^ + MAs^V^), and total dimethylarsenic (DMAs = DMAs^III^ + DMAs^V^) species were determined. We confirmed the identities of arsenicals in spectral peaks using aliquots of samples spiked with standards. Calibration curves for each of the arsenicals (0.5, 2.5, 10, 20, 80 ng As) were generated to quantify results of the analyses.

### Immunoblot analyses

Protein extracts were prepared from cells treated with arsenicals, activated with insulin, and incubated with d-glucose, using a 25-mM HEPES (pH 7.4) lysis buffer containing 1% NP 40, 100 mM NaCl, 2% glycerol, 5 mM sodium fluoride, 1 mM EDTA, 1 mM sodium orthovanadate (Na_3_VO_4_), 1 mM sodium pyrophosphate, 1 mM PMSF, 40 μg/mL aprotinin, 20 μg/mL leupeptin, and 20 μg/mL pepstatin (all from Sigma Chemical Co.). Protein extracts were separated by 10% SDS-PAGE, electroblotted to Immobilon-P membranes (Millipore, Burlington, MA), and probed using the following antibodies: anti-p38 MAPK (mitogen-activated protein kinase), anti-p-p38 MAPK, anti-PKB/Akt, anti-p-PKB/Akt(Ser473) and anti-p-PKB/Akt(Thr308), anti-PTEN (phosphatase and tensin homolog deleted on chromosome ten), anti-p-PTEN(Ser380), anti-p-PDK-1(Ser241) (Cell Signaling Technology, Beverly, MA); anti-PI-3K(p85) (Upstate Biotechnology, Lake Placid, NY); and anti-β-actin (Abcam, Cambridge, MA). The antigen-antibody complexes on immunoblots were treated with horseradish peroxidase–conjugated secondary antibodies (Santa Cruz Biotechnology) and visualized using autoradiography or the Gene Gnome imaging system (Syngene, Frederick, MD).

### Protein kinase activity assays

We measured PI-3K, PKB/Akt, and PDK-1 activities in cell lysates from adipocytes treated with arsenicals and activated with insulin after immunoprecipitation with specific antibodies bound to protein G agarose beads (Santa Cruz Biotechnology). For a single assay we used the immunoprecipitate from cells cultured in one 10-cm plate. The assay conditions were as follows:

#### PI-3K assay

PI-3K was immunoprecipitated from control (untreated) adipocytes or from adipocytes exposed to arsenicals with an anti-phosphotyrosine (PY20) antibody. PI-3K immunoprecipitated from insulin-activated adipocytes pretreated with 1 nM wortmannin (a specific inhibitor of PI-3K) was used as a negative control. The enzyme activity was measured in a 50-μL assay mixture containing the immunoprecipitated PI-3K, 20 mM HEPES (pH 7.4), 50 mM MgCl_2_, 200 μM adenosine, 40 μM adenosine 5′-triphosphate (ATP) (all from Sigma Chemical Co.), 20 μCi [γ-^32^P]-ATP (NEN Life Science Products, Inc.), and l-α-phosphatidylinositol (PI) (Avanti Polar Lipids Inc., Alabaster, AL) as a substrate (Augustine et al. 1991). The reaction was stopped by 1 N HCl. A 30-min incubation at 37°C of the assay mixture containing PI-3K from control cells resulted in the formation of radiolabeled phosphatidylinositol phosphate (PIP) and phosphatidylinositol bisphosphate (PIP_2_). To simplify the analysis, a 10-min incubation that yielded only PIP was used throughout this study. Radiolabeled phospholipids were extracted in chloroform:methanol (CHCl_3_:CH_3_OH) (1:1). The organic phase was washed with CH_3_OH:HCl (1:1), evaporated under nitrogen, and the residue was dissolved in CHCl_3_:CH_3_OH (2:1). The extract was separated by thin-layer chromatography (TLC) on glass silica plates pretreated with 1% potassium oxalate, using an *n*-propanol:2 N acetic acid (65:35) solvent system (Augustine et al. 1991). The distribution of radioactivity on TLC plates was evaluated, using a computerized Fuji FLA-2000 imaging system (Fujifilm, Stamford, CT). The following standards were used to confirm the identity of separated phospholipids: l-α-phosphatidylinositol, l-α-phosphatidylinositol-4-phosphate, l-α-phosphatidylinositol-4,5-bisphosphate (Avanti Polar Lipids Inc.). Standards were visualized on developed TLC plates by treatment with 50% sulfuric acid at 100°C for 1 hr.

#### PKB/Akt assay

We measured PKB/Akt activity using an Akt1/PKBα Immunoprecipitation-Kinase Assay Kit (Upstate Biotechnology), following the manufacturer’s protocol. PKB/Akt was immunoprecipitated with an antibody raised against the pleckstrin homology domain of PKB/Akt. The assay mixture contained 20 mM morpholine-propanesulfonic acid (pH 7.2), 25 mM β-glycerophosphate, 5 mM EGTA, 1 mM Na_3_VO_4_, 1 mM dithiothreitol, 10 mM protein kinase A (PKA) inhibitor peptide (Upstate Biotechnology), 19 mM MgCl_2_, 125 μM ATP, 5 μCi [γ-^32^P]-ATP, and Akt/SGK peptide as a substrate. Incubation was carried out at 30°C for 10 min with continuous shaking. The radiolabeled peptide was blotted on P81 phosphocellulose and quantified by liquid scintillation.

#### PDK-1 assay

PDK-1 activity was measured using a PDK-1 Kinase Assay Kit (Upstate Biotechnology). PDK-1 was immunoprecipitated with an anti-p-PDK-1(Ser241) antibody (Cell Signaling Technology). The assay mixture contained 50 mM Tris–HCl (pH 7.5), 0.1 mM EGTA, 0.1 mM EDTA, 1 mM Tris(2-carboxyethyl) phosphine, 25 μM PKA peptide inhibitor (Upstate Biotechnology), 1 μM microcystin-LR, 10 mM magnesium acetate, 15 mM MgCl_2_, 100 μM ATP and 5 μCi [γ-^32^P]-ATP. The assay was performed at 30°C in two incubation steps. In the first 30-min step, PDK-1 phosphorylates (activates) recombinant serum and glucocorticoid-induced protein kinase-1 (SGK1). In the second 10-min step, the activated SGK1 phosphorylates a synthetic peptide (RPRAATF) using [γ-^32^P]-ATP as a phosphate donor. The radiolabeled peptide is blotted on P81 phosphocellulose and quantified by liquid scintillation.

### Statistical analysis

All experiments were replicated to ensure the reproducibility of results. Representative findings are shown. Results of the cell viability, glucose uptake, and protein kinase activity assays were evaluated by analysis of variance with Tukey multiple comparison posttest using a GraphPad Instat statistical software package (GraphPad Software, San Diego, CA). Differences among means with *p* < 0.05 were considered statistically significant.

## Results

**Figure 1:**
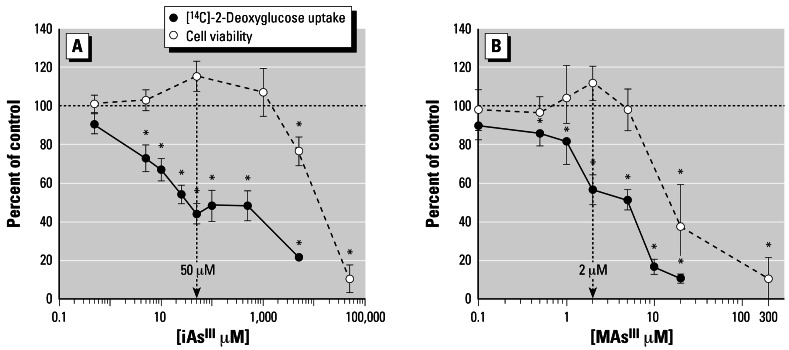
iAs^III^ and MAs^III^ inhibit ISGU by adipocytes at concentrations that do not compromise cell viability. 3T3-L1 adipocytes were treated with iAs^III^ (*A*) or MAs^III^ (*B*) for 4 hr. [^14^C]-2-Deoxyglucose uptake was assessed in treated and untreated (control) cells after a 10-min activation with insulin. Cell viability was measured by MTT assay. Each value represents the mean ± SD; *n* = 3–5 experiments. *Statistically significant differences (*p* < 0.05) between treated and control cells. The concentrations of 50 μM iAs^III^ and 2 μM MAs^III^ (indicated by arrows) were used in all subsequent experiments.

**Figure 2:**
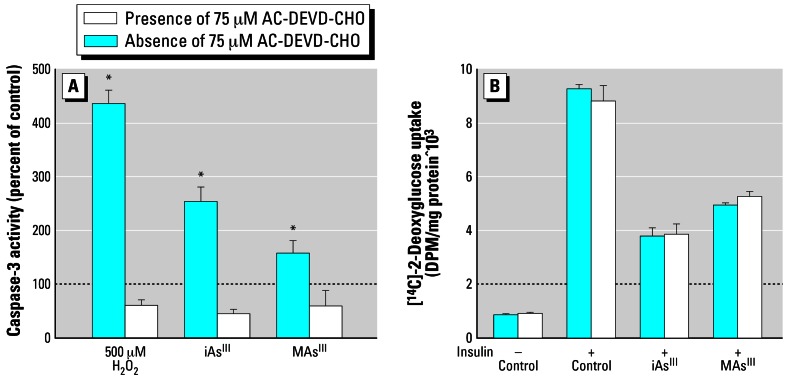
ISGU in adipocytes exposed to subtoxic concentrations of iAs^III^ and MAs^III^ is independent of caspase-3 activation. (*A*) Caspase-3 activity was measured in 3T3-L1 adipocytes treated for 4 hr with 50 μM iAs^III^ or 2 μM MAs^III^ and in untreated (control) cells in the presence or absence of 75 μM AC-DEVD-CHO, a caspase-3 inhibitor. Adipocytes treated with 500 μM H_2_O_2_ for 4 hr were used as positive controls. (*B*) Basal and insulin-stimulated uptake of [^14^C]-2-deoxyglucose was measured in adipocytes exposed for 4 hr to 50 μM iAs^III^ or 2 μM MAs^III^ in the presence or absence of 75 μM AC-DEVD-CHO. Each value represents the mean ± SD; *n* = 3–5 experiments. *Statistically significant differences (*p* < 0.05) between treated and untreated (control) cells.

**Figure 3:**
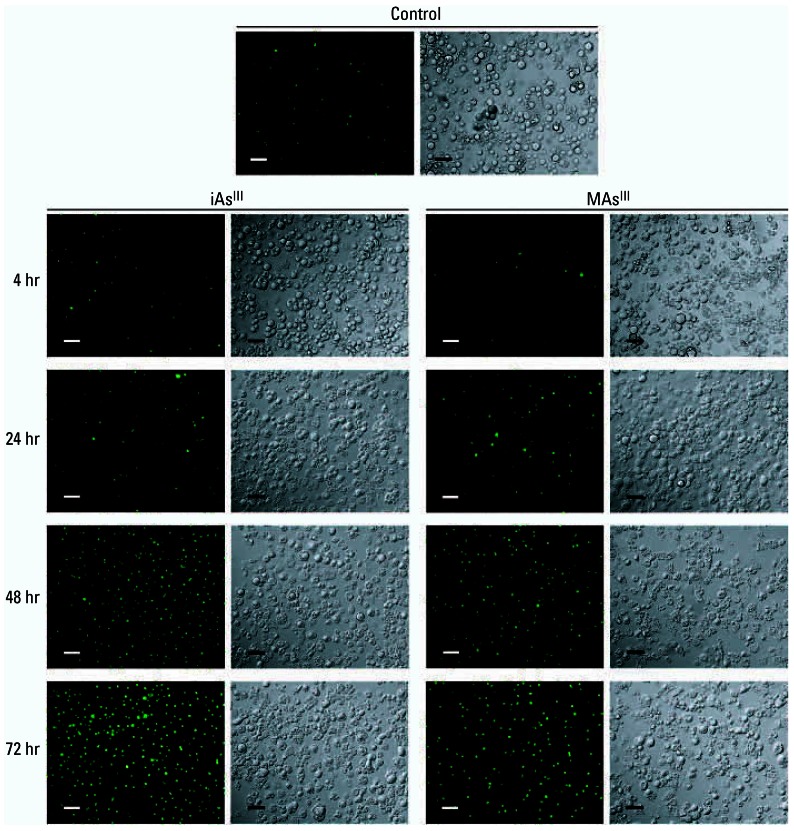
Four-hour exposures to subtoxic concentrations of iAs^III^ or MAs^III^ do not increase DNA fragmentation in cultured adipocytes. DNA fragmentation was measured by TUNEL in 3T3-L1 adipocytes treated with 50 μM iAs^III^ or 2 μM MAs^III^ for 4, 24, 48, and 72 hr. Untreated adipocytes were used as controls. Color images show green fluorescein signal of fragmented DNA in apoptotic cells. Gray-scale images illustrate the corresponding cell morphology. Representative fields of two independent experiments are shown. Bars = 40 μm.

Our previous work has shown that trivalent arsenicals inhibit ISGU by 3T3-L1 adipocytes. However, the possible association between the inhibition of ISGU and a general loss of cell functions due to the cytotoxicity of arsenicals has not been thoroughly examined. In this study we examined ISGU and cell viability in adipocytes exposed for 4 hr to iAs^III^ or MAs^III^ at a wide range of concentrations. Consistent with our previous report (Walton et al. 2004), stimulation of 3T3-L1 adipocytes with insulin increased glucose uptake by 9- to 11-fold over basal levels (data not shown). ISGU was significantly inhibited by concentrations as low as 5 μM iAs^III^ and 0.5 μM MAs^III^ (Figure 1A, B). In contrast, cell viability decreased only when concentrations of iAs^III^ and MAs^III^ exceeded 1 mM and 5 μM, respectively. Gross abnormalities in adipocyte morphology were absent at all concentrations tested, although minor cell detachment did occur at higher concentrations (≥ 200 μM iAs^III^ and ≥ 10 μM MAs^III^). The estimated IC_50_ (concentration that results in the inhibition of ISGU by 50%) values for the inhibition of ISGU were 25 μM for iAs^III^ and 4 μM for MAs^III^. In comparison, the LC_50_ (concentration that results in a decrease of cell viability by 50%) values characterizing the cytotoxic effects were 11 mM for iAs^III^ and 15 μM for MAs^III^. Thus, the inhibition of ISGU by iAs^III^ and MAs^III^ at or below IC_50_ values was not due to impaired adipocyte viability. However, both iAs^III^ and MAs^III^ can induce cell apoptosis (Lau et al. 2004; McCollum et al. 2005; Namgung and Xia 2001). At early stages, apoptotic processes may affect cell functions without having immediate effects on cell viability. We examined apoptotic markers in adipocytes exposed for 4 hr to 50 μM iAs^III^ and 2 μM MAs^III^, the concentrations that effectively inhibit ISGU, but are far below the minimal cytotoxic concentrations. Under these exposure conditions, both iAs^III^ and MAs^III^ significantly increased the activity of caspase-3, an early marker of apoptosis (Figure 2A). Adipocytes treated with 500 μM H_2_O_2_ were used as positive controls for this experiment. Pretreatment with 75 μM Ac-Asp-Glu-Val-Asp-CHO (AC-DEVD-CHO), a cell-permeable caspase-3 inhibitor, prevented caspase-3 activation by both arsenicals and by hydrogen peroxide. However, pre-treatment with AC-DEVD-CHO did not prevent the decrease in ISGU in cells treated with either iAs^III^ or MAs^III^ (Figure 2B), suggesting that the inhibition of ISGU was independent of processes associated with early stages of apoptosis. TUNEL was used to determine the degree of DNA fragmentation in adipocytes exposed to 50 μM iAs^III^ or 2 μM MAs^III^ (Figure 3). Adipocyte nuclei were stained with DAPI to determine the total number of cells (data not shown). The average apoptotic index (percentage of TUNEL-positive cells) was about 16% for control adipocytes and did not change after a 4-hr exposure to either iAs^III^ or MAs^III^. However, the apoptotic index increased considerably after longer exposure times, reaching an average of 32% for iAs^III^ and 39% for MAs^III^ after 24 hr and more than 90% after 72-hr exposure to either arsenical. These data suggest that 4-hr exposures to 50 μM iAs^III^ or 2 μM MAs^III^ did not compromise cell viability or integrity. In addition, neither 50 μM iAs^III^ nor 2 μM MAs^III^ induced p38 MAPK phosphorylation during the 4-hr exposure (data not shown). Therefore, the inhibition of ISGU is not associated with stress and is likely due to specific effects of these arsenicals on mediators of insulin signaling or on the cellular components involved in glucose transport. Based on these results, 4-hr exposures to 50 μM iAs^III^ and 2 μM MAs^III^ were used in further experiments to examine the effects of iAs^III^ or MAs^III^ on components of the insulin-activated signal transduction pathway in 3T3-L1 adipocytes.

**Figure 4:**
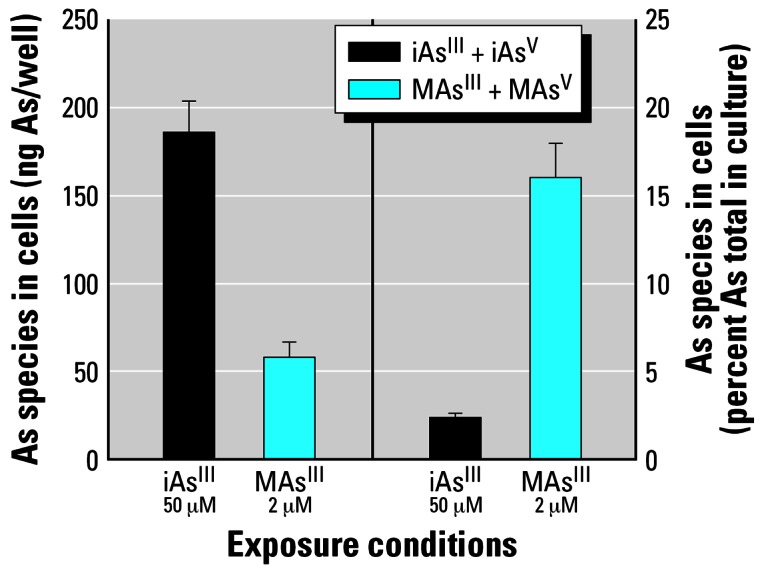
Retention of As species in 3T3-L1 adipocytes exposed to iAs^III^ or MAs^III^. As species retained in 3T3-L1 adipocytes exposed for 4 hr to 50 μM iAs^III^ or 2 μM MAs^III^ were analyzed by HG-AAS. Note: The HG-AAS technique used in this study cannot distinguish between As^III^ and As^V^ species. Each value represents the mean ± SD; *n* = 3 experiments.

The effects of iAs^III^ or MAs^III^ on mediators of insulin signaling would ultimately depend on the intracellular concentrations and metabolic conversion of these arsenicals. We examined the distribution of As species in adipocytes after a 4-hr exposure to iAs^III^ or MAs^III^, using HG-AAS. Cells exposed to 50 μM iAs^III^ retained about 3 times more As than cells exposed to 2 μM MAs^III^ (Figure 4). Retained As represented 2.5 and 16% of the total As in cultures exposed to iAs^III^ or MAs^III^, respectively. Only iAs and MAs species were detected in adipocyte cultures exposed to iAs^III^ and MAs^III^, respectively, indicating that no methylation conversion took place during the 4-hr exposures. These findings are consistent with previous reports that found adipocytes to be inefficient methylators of iAs (Walton et al. 2004).

**Figure 5:**
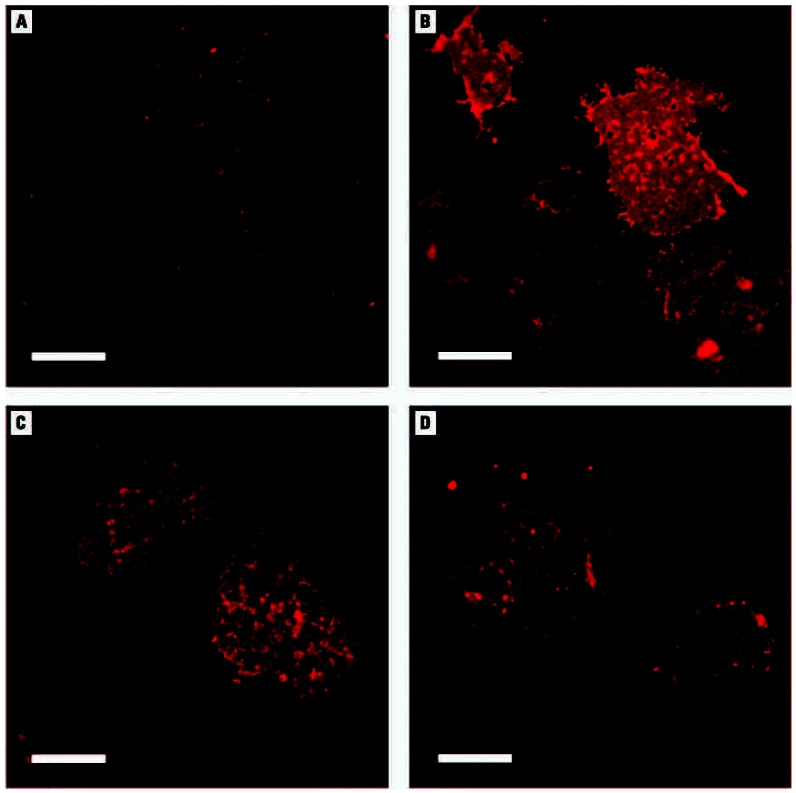
Exposures to subtoxic concentrations of iAs^III^ or MAs^III^ inhibit GLUT4 association with the plasma membrane of insulin-activated adipocytes. Immunofluorescent images of GLUT4 in plasma membrane lawns isolated from control (untreated) 3T3-L1 adipocytes before (*A*) and after activation (*B*) with insulin and from insulin-activated adipocytes treated for 4 hr with 50 μM iAs^III^ (*C*) or 2 μM MAs^III^ (*D*). Adipocytes were fixed and sonicated to prepare plasma membrane lawns. GLUT4 was labeled with an anti-GLUT4 antibody and visualized with a fluorescent secondary antibody. Representative fields of two independent experiments are shown. Bars = 10 μm.

The translocation of GLUT4 from the perinuclear compartment to the plasma membrane is a prerequisite for glucose uptake in adipocytes stimulated with insulin. We used immunofluorescent staining in this study to examine the association of GLUT4 with the plasma membranes of insulin-stimulated 3T3-L1 adipocytes treated with 50 μM iAs^III^ or 2 μM MAs^III^ for 4 hr and from control (untreated) cells that were or were not stimulated with insulin (Figure 5). Stimulation with insulin dramatically increased the GLUT4-specific fluorescent signal in plasma membrane lawns of control cells. GLUT4 signals in plasma membrane lawns isolated from insulin-stimulated cells treated with either iAs^III^ or MAs^III^ were noticeably weaker compared with control insulin-stimulated cells, suggesting that both arsenicals interfered with the translocation of GLUT4 in response to insulin stimulation.

**Figure 6:**
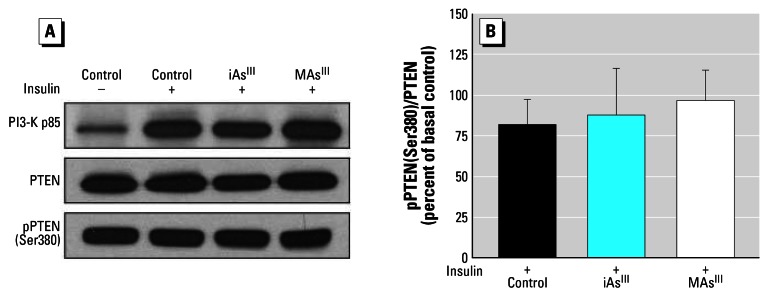
Exposures to subtoxic concentrations of iAs^III^ or MAs^III^ do not affect insulin signal mediators that regulate PIP_3_ levels in insulin-activated adipocytes. (*A*) Immunoblot analyses of the activated PI-3K, total PTEN, and phosphorylated PTEN (Ser380) in control 3T3-L1 adipocytes before or after activation with insulin and in insulin-activated adipocytes treated for 4 hr with 50 μM iAs^III^ or 2 μM MAs^III^. Activated PI-3K was immunoprecipitated from control and exposed cells with an anti-phospho-Tyr (PY20) antibody and immunoblotted with an antibody against the regulatory (p85) subunit. Representative blots of three independent experiments are shown. (*B*) The ratio of phosphorylated PTEN (Ser380) to total PTEN expressed as a percent of the ratio found in control adipocytes before activation with insulin. Each value represents the mean ± SD; *n* = 3 experiments.

The impaired ISGU in adipocytes exposed to trivalent arsenicals has previously been linked to the inhibition of components of the insulin signal transduction pathway located downstream of IRS1/2, but upstream of PKB/Akt (Walton et al. 2004). PI-3K is located downstream of IRS. The binding of p-IRS to the regulatory (p85) subunit of PI-3K in response to insulin stimulates the PI-3K–catalyzed production of PIP_3_ from PIP_2_. In this study, the association of p-IRS with PI-3K was examined in insulin-stimulated adipocytes exposed for 4 hr to 50 μM iAs^III^ or 2 μM MAs^III^. Neither iAs^III^ nor MAs^III^ affected the amount of PI-3K (p85), immunoprecipitated with an anti-phosphotyrosine (PY20) antibody, which reacts with phosphorylated tyrosine residues of IRS in the insulin-activated PI-3K complex (Figure 6A). PI-3K activity was measured in adipocytes exposed for 4 hr to 50 or 100 μM iAs^III^ or to 2 or 5 μM MAs^III^. Exposures to iAs^III^ had no effect on PI-3K activity. A relatively small decrease in PI-3K activity was detected in cells exposed to 2 μM MAs^III^; however, no changes were found in cells exposed to 5 μM MAs^III^ (data not shown). Effects of MAs^III^ on PI-3K activity were further analyzed in an *in vitro* assay mixture containing PI-3K immunoprecipitated from control insulin-stimulated adipocytes. Addition of MAs^III^ into this mixture at concentrations up to 50 μM did not inhibit PI-3K activity (data not shown). PTEN, a PIP_3_ phosphatase, is involved in the regulation of PIP_3_ levels in adipocytes. PTEN activity is regulated by a casein kinase 2–catalyzed phosphorylation on its C-terminal non-catalytic regulatory domain, which includes Ser380 (Torres and Pulido 2001). Neither 50 μM iAs^III^ nor 2 μM MAs^III^ altered the levels of total PTEN or pPTEN (Ser380) (Figure 6A). No changes in the of ratio of phosphorylated pPTEN (Ser380) to total PTEN were found in insulin-stimulated adipocytes exposed to either iAs^III^ or MAs^III^ (Figure 6B).

**Figure 7:**
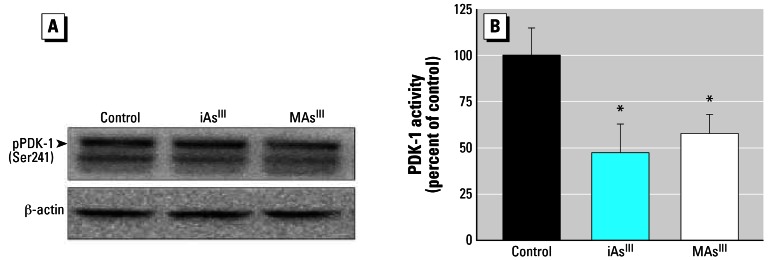
Exposures to subtoxic concentrations of As^III^ or MAs^III^ do not affect the phosphorylation of PDK-1 but inhibit PDK-1 activity in adipocytes. (*A*) Immunoblot analysis of Ser241-phosphorylated PDK-1 in insulin-activated 3T3-L1 adipocytes treated for 4 hr with 50 μM iAs^III^ or 2 μM MAs^III^ and in control (untreated) insulin-activated adipocytes. Representative blot of three independent experiments is shown. (*B*) The kinase activity of PDK-1 immunoprecipitated from insulin-activated control or treated 3T3-L1 adipocytes. Each value represents the mean ± SD; *n* = 4–5 experiments. *Statistically significant differences (*p* < 0.05) between treated and control cells.

Phosphorylation on Ser241 is required for optimal activity of PDK-1, a downstream effector of PI-3K (Casamayor et al. 1999). Figure 7A shows that exposures to 50 μM iAs^III^ or 2 μM MAs^III^ had no significant effects on the level of Ser241-phosphorylated PDK-1 in insulin-stimulated adipocytes. However, PDK-1 activity was significantly lower in cells exposed to either iAs^III^ or MAs^III^, 47% and 57% of that in control cells, respectively (Figure 7B).

**Figure 8:**
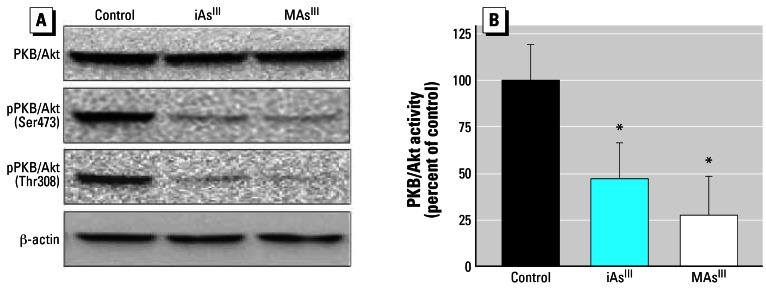
Exposures to subtoxic concentrations of iAs^III^ or MAs^III^ inhibit the phosphorylation and PKB/Akt. (*A*) Immunoblot analysis of total PKB/Akt and Ser473- or Thr308-phosphorylated PKB/Akt in insulin-activated 3T3-L1 adipocytes treated for 4 hr with 50 μM iAs^III^ or 2 μM MAs^III^ and in insulin-activated control (untreated) cells. Representative blots of three independent experiments are shown. (*B*) The activity of PKB/Akt immunoprecipitated from insulin-activated control and treated adipocytes. Each value represents the mean ± SD; *n* = 4–5 experiments. *Statistically significant differences (*p* < 0.05) between treated and control cells.

In the insulin-activated signal transduction pathway, PKB/Akt is the downstream effector of PDK-1. The activation of PKB/Akt in response to insulin stimulation includes the phosphorylation of Ser473 and Thr308 residues (Toker and Newton 2000). Our previous work demonstrated that exposures to iAs^III^ or MAs^III^ inhibit PKB/Akt phosphorylation on Ser473 (Walton et al. 2004), which is thought to be catalyzed by a putative Ser-kinase, PDK-2 (Toker and Newton 2000). PDK-1 is responsible for Thr308 phosphorylation, which is required for maximal PKB/Akt activity (Scheid et al. 2002). Immunoblot analysis carried out in this study showed that 4-hr exposures to 50 μM iAs^III^ or 2 μM MAs^III^ inhibited the insulin-dependent phosphorylation of PKB/Akt on both Ser473 and Thr308 residues (Figure 8A). PKB/Akt activity in insulin-stimulated adipocytes exposed to iAs^III^ and MAs^III^ was 47 and 28% of that in control insulin-activated cells, respectively (Figure 8B).

**Figure 9:**
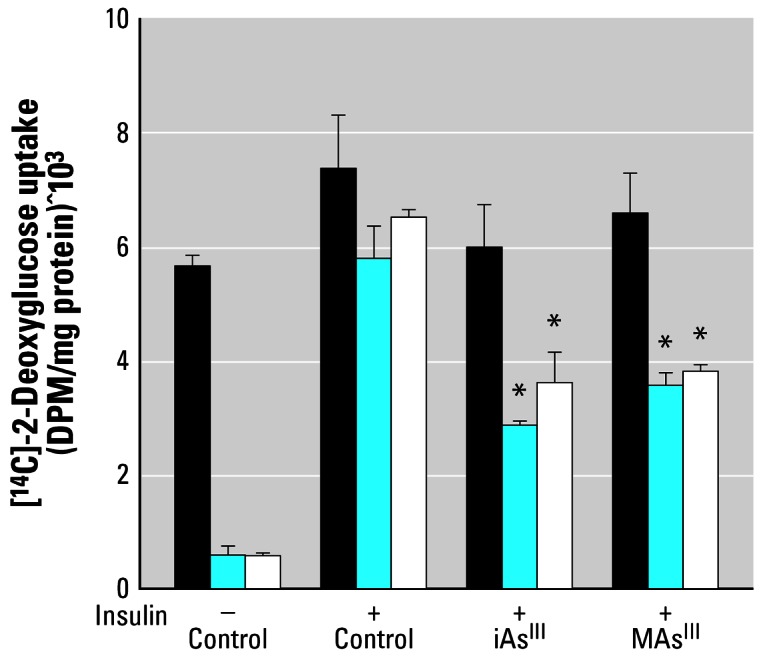
Constitutive activation of protein kinase B (PKB/Akt) prevents the inhibition of insulin-stimulated glucose uptake in adipocytes treated with subtoxic concentrations of iAs^III^ or MAs^III^. Basal and insulin-stimulated [^14^C]-2-deoxyglucose uptake by 3T3-L1 adipocytes treated for 4 hr with 50 μM iAs^III^ or 2 μM MAs^III^ and by untreated adipocytes expressing a constitutively active myr-PKB/Akt) (black bar, an inactive PKB/Akt mutant (A2myr-PKB/Akt) (blue bar), or an empty expression vector (white bar). Each value represents the mean ± SD; *n* = 4–5 experiments. *Statistically significant differences (*p* < 0.05) between treated and control cells.

To further evaluate the role of the PDK-1/PKB/Akt signal transduction step as a target for trivalent arsenicals in the insulin-activated signal transduction pathway, we examined the effects of iAs^III^ or MAs^III^ on ISGU by adipocytes expressing constitutively active myr-PKB/Akt. Adipocytes expressing an inactive A2myr-PKB/Akt mutant or empty expression vector were used as negative controls. Consistent with the constitutive activation of PKB/Akt, glucose uptake by adipocytes expressing myr-PKB/Akt was elevated even in the absence of insulin stimulation (Figure 9). Four-hour exposures to 50 μM iAs^III^ or 2 μM MAs^III^ had no effect on ISGU by myr-PKB/Akt expressing cells. In contrast, both arsenicals inhibited ISGU in cells expressing the inactive A2myr-PKB/Akt mutant or the empty expression vector.

## Discussion

Previous studies have shown that As^III^-containing species may affect glucose uptake by cultured cells or dissected tissues by two independent mechanisms that strictly depend on the concentration of As^III^. Highly-toxic concentrations of As^III^ stimulate glucose uptake in the absence of insulin (Bazuine et al. 2003, 2004; Brazy et al. 1980; McDowell et al. 1997; Pasternak et al. 1991; Short 1965; Sviderskaya et al. 1996; Widnell et al. 1990) through a mechanism that involves activation of p38 MAPK-mediated stress signaling and PI-3K-dependent phosphorylation of PKB/Akt (Souza et al. 2001). In our experiments, exposure of 3T3-L1 adipocytes to 50 μM iAs^III^ and 2 μM MAs^III^ for 4 hr did not activate p38 MAPK, thus providing further evidence of the subtoxic nature of our exposure conditions. Treatments with toxic concentrations of arsenicals are not comparable to environmental or occupational exposures to iAs that do not typically induce acute stress or tissue damage. In contrast, subtoxic concentrations of As^III^ inhibit ISGU (Henriksen and Holloszy 1990; Liebl et al. 1992, 1995; Sowell et al. 1988) in a manner consistent with impaired glucose tolerance reported among individuals chronically exposed to relatively low concentrations of iAs. We have shown that inhibition of ISGU in adipocytes exposed to subtoxic concentrations of trivalent metabolites of iAs, iAs^III^, or MAs^III^ is associated with the suppression of PKB/Akt phosphorylation (Walton et al. 2004). Because neither iAs^III^ nor MAs^III^ interfered with insulin signaling upstream of PI-3K (Walton et al. 2004), the present work focused on the signal transduction steps immediately preceding PKB/Akt phosphorylation, specifically, on the enzymatic system controlling PIP_3_ levels in insulin-activated cells and on PDK-1.

The formation of PIP_3_ catalyzed by the insulin-activated PI-3K-IRS complex is an essential step in ISGU by adipocytes. PIP_3_ is required for PDK-1–catalyzed phosphorylation of PKB/Akt on Thr308 (Casamayor et al. 1999). PIP_3_ is thought to interact directly with the PH-domain of PDK-1 and PKB/Akt, activating PDK-1 or facilitating Thr308 phosphorylation of PKB/Akt. Other studies suggest that PIP_3_ promotes the phosphorylation of PKB/Akt on Ser473 by a putative PDK-2, thereby priming PKB/Akt for PDK-1–catalyzed phosphorylation of Thr308 (Toker and Newton 2000). PIP_3_ concentration in the membrane region of cells is subjected to strict regulation involving PI-3K and specific lipid phosphatases, including PTEN (Maehama and Dixon 1998) and SHIP2 (Src homology 2-containing inositol 5′-phosphatase 2) (Wada et al. 2001). Both PTEN, a D-3 lipid phosphatase, and SHIP2, a D-5 lipid phosphatase, are expressed in adipocytes. However, a recent report suggests that only PTEN is capable of suppressing insulin signaling in 3T3-L1 adipocytes (Tang et al. 2005). PTEN is phosphorylated on Ser380 and Thr382/383 by casein kinase 2 (Torres and Pulido 2001). The phosphorylated PTEN (p-PTEN) is less susceptible to degradation by the proteosome but is less active. Inhibition of Ser380 phosphorylation increases PTEN activity, but destabilizes the enzyme (Georgescu et al. 1999; Tolkacheva and Chan 2000). Factors that interfere with PI-3K activation in response to insulin or inhibit PTEN phosphorylation may decrease PIP_3_ levels in adipocytes and, ultimately, prevent PDK-1/2–catalyzed phosphorylation of PKB/Akt.

In this study, iAs^III^ and MAs^III^ inhibited PDK-1/2 catalyzed phosphorylation of PKB/Akt on Thr308 and Ser473 but had little or no effect on PI-3K activity or PTEN phosphorylation. In addition, neither iAs^III^ nor MAs^III^ affected PDK-1(Ser241) phosphorylation, which is essential for PDK-1 activity. These results suggest that iAs^III^ and MAs^III^ inhibit PDK-1 activity through direct interactions with the enzyme. Sulfhydryl groups of vicinal or closely spaced cysteines are typical high-affinity targets for trivalent arsenicals in protein structures (Altamirano et al. 1989; Carlson et al. 1978; Chakraborti et al. 1992; Delnomdedieu et al. 1993; Li et al. 2001; Lopez et al. 1990). Two such closely spaced cysteines (Cys21 and Cys23) are present in the N-terminus of both mouse and human PDK-1 (Alessi et al. 1997; Dong et al. 1999). Thus, it is plausible that binding of iAs^III^ and MAs^III^ to Cys21 and Cys23 is the proximate cause of PDK-1 inhibition by these arsenicals. However, unlike MAs^III^, which can form a stable cyclic structure with two thiols, iAs^III^ may require three coordination bonds to form a stable enzyme-inhibitor complex. A lower affinity for binding to Cys21 and Cys23 may explain why iAs^III^ is a weaker inhibitor of PDK-1 than MAs^III^. In addition, the difference in potencies of iAs^III^ and MAs^III^ to inhibit PDK-1 activity and ISGU may be in part due to differences in the uptake and/or retention of these arsenicals by adipocytes. Our data suggest that MAs^III^ was retained by 3T3-L1 adipocytes more efficiently than iAs^III^. These findings are consistent with the results of previous studies in other cell types (Dopp et al. 2004; Drobna et al. 2005). Importantly, our data show that the expression of constitutively active myrPKB/Akt prevents the inhibition of ISGU by either iAs^III^ or MAs^III^. These data provide further evidence that the inhibition of ISGU by 3T3-L1 adipocytes exposed to iAs^III^ and MAs^III^ is due to the inhibition of the PDK-1–catalyzed activation of PKB/Akt and that neither iAs^III^ nor MAs^III^ disrupts signal transduction steps downstream from PDK-1/PKB/Akt, or events associated with GLUT4 translocation to the plasma membrane.

**Figure 10:**
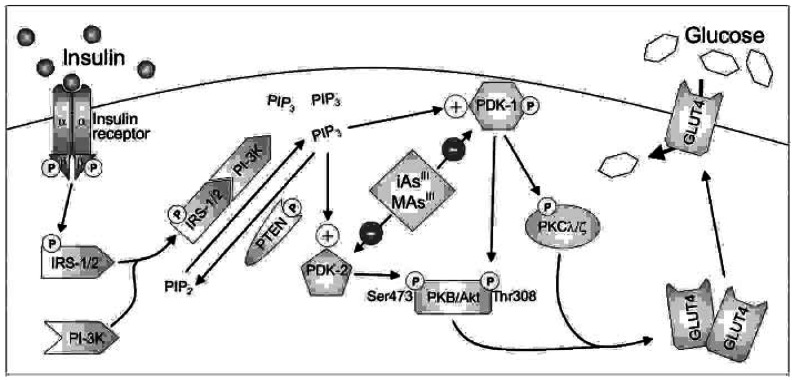
The molecular mechanism underlying the inhibition of insulin-stimulated glucose uptake by adipocytes exposed to iAs^III^ or MAs^III^. The inhibition of PDK-1 and putative PDK-2 activities by iAs^III^ and MAs^III^ results in suppression of the downstream signaling steps, including PKB/Akt phosphorylation and GLUT4 translocation to the plasma membrane.

In summary, subtoxic concentrations of iAs^III^ and MAs^III^ inhibit ISGU by 3T3-L1 adipocytes through a mechanism that involves the inhibition of PDK-1 activity and of PDK-1/2–catalyzed phosphorylation of PKB/Akt (Figure 10). The inhibition of ISGU by iAs^III^ and MAs^III^, trivalent metabolites of iAs, is consistent with impaired glucose tolerance reported in individuals chronically exposed to iAs from the environment. In addition, the concentrations of iAs^III^ and MAs^III^ that inhibit ISGU by cultured adipocytes (as low as 5 and 0.5 μM, respectively) appear to be compatible with this type of exposure. Thus, taken together, this work provides a mechanistic basis for the diabetogenic effects of chronic environmental and occupational exposures to iAs.
